# High prevalence of p16 genetic alterations in head and neck tumours

**DOI:** 10.1038/sj.bjc.6690747

**Published:** 1999-10

**Authors:** E C Miracca, L P Kowalski, M A Nagai

**Affiliations:** Disciplina de Oncologia, Departamento de Radiologia, FMUSP, Av Dr Arnaldo 455, 4 andar, São Paulo, CEP, 01296-903, Brazil; Fundação Antônio Prudente, Rua Antônio Prudente 211, São Paulo, CEP, 01509-900, Brazil

**Keywords:** p16, head and neck tumours, hypermethylation, LOH

## Abstract

Inactivation of the p16 gene is believed to contribute to the tumorigenic process of several neoplasms, including head and neck tumours. In the present study, DNA samples from paired tumour and adjacent normal tissue from 47 patients with squamous cell carcinoma of the head and neck were investigated for the occurrence of p16 genetic alterations. Single-strand conformation polymorphism and direct DNA sequence analysis led to the identification of p16 mutations in six cases (13%). Southern blot analysis showed that homozygous deletion is a rare event in the group of tumours analysed. Loss of heterozygosity (LOH) analysis was performed by polymerase chain reaction (PCR) using two microsatellite markers (IFNA and D9S171) from the 9p21 region. Taking into account only the informative cases, 17 of 32 tumours (53%) showed LOH for at least one of the markers analysed. The methylation status of the CpG sites in the exon 1 of the p16 gene was analysed using methylation-sensitive restriction enzymes and PCR amplification. Hypermethylation was observed in 22 (47%) of the head and neck tumours analysed. In our series of head and neck tumours, evidence for inactivation of both p16 alleles was observed in 13 cases with hypermethylation and LOH, two cases with hypermethylation and mutation, four cases with mutation and LOH and one case with homozygous deletion. These findings provide further evidence that genetic alterations, especially hypermethylation and LOH, leading to the inactivation of the p16 tumour suppressor gene are common in primary head and neck tumours. © 1999 Cancer Research Campaign


*British Journal of Cancer* (1999) **81**(4), 677–683
© 1999 Cancer Research Campaign
Article no. bjoc.1999.0747

### High prevalence of p16 genetic alterations in head and neck tumours

**EC Miracca[1], LP Kowalski[2] and MA Nagai[1]**

[1]Disciplina de Oncologia, Departamento de Radiologia, FMUSP, Av Dr Arnaldo 455, 4 andar, São Paulo, CEP 01296-903, Brazil; [2]Fundação Antônio Prudente, Rua Antônio Prudente 211, São Paulo, CEP 01509-900, Brazil

**Summary** Inactivation of the p16 gene is believed to contribute to the tumorigenic process of several neoplasms, including head and neck tumours. In the present study, DNA samples from paired tumour and adjacent normal tissue from 47 patients with squamous cell carcinoma of the head and neck were investigated for the occurrence of p16 genetic alterations. Single-strand conformation polymorphism and direct DNA sequence analysis led to the identification of p16 mutations in six cases (13%). Southern blot analysis showed that homozygous deletion is a rare event in the group of tumours analysed. Loss of heterozygosity (LOH) analysis was performed by polymerase chain reaction (PCR) using two microsatellite markers (IFNA and D9S171) from the 9p21 region. Taking into account only the informative cases, 17 of 32 tumours (53%) showed LOH for at least one of the markers analysed. The methylation status of the CpG sites in the exon 1 of the p16 gene was analysed using methylation-sensitive restriction enzymes and PCR amplification. Hypermethylation was observed in 22 (47%) of the head and neck tumours analysed. In our series of head and neck tumours, evidence for inactivation of both p16 alleles was observed in 13 cases with hypermethylation and LOH, two cases with hypermethylation and mutation, four cases with mutation and LOH and one case with homozygous deletion. These findings provide further evidence that genetic alterations, especially hypermethylation and LOH, leading to the inactivation of the p16 tumour suppressor gene are common in primary head and neck tumours. © 1999 Cancer Research Campaign

**Keywords**: p16; head and neck tumours; hypermethylation; LOH

The *p16* tumour suppressor gene located on chromosome 9p21, encodes a 16 kDa protein that acts as a cyclin-dependent kinase (cdk) 4/6 inhibitor (Serrano et al, 1993). This gene, whose locus is denominated CDKN2A, has also been named *MTS1* and $p16^{ink4a}$ (Kamb et al, 1994*a*; Ranade et al, 1995). The CDKN2A gene utilizes alternative first exons and common downstream exons to encode two structurally unrelated proteins, $p16^{ink4a}$ and $p19^{arf}$, which mediate cell cycle arrest through different mechanisms (Quelle et al, 1995; Stone et al, 1995; Chin et al, 1998).

The progression of proliferating cells through the different phases of the cell cycle is highly regulated by activators and inhibitors (Hunter and Pines, 1994). p16 belongs to an important group of proteins that includes the $p15^{ink4b}$, $p21^{waf1}$ and $p27^{kip1}$, which negatively regulate the G1 phase of the cell cycle (Serrano et al, 1993). The p16 gene product binds to cdk4 and cdk6 inhibiting their association with cyclin D1. The inhibition of the cyclin D1–cdk4/6 complex activity prevents retinoblastoma protein (pRB) phosphorylation and the release of E2F, leading to the inhibition of the cell cycle in the G1/S transition (Serrano et al, 1993; Tam et al, 1994; Yeundall and Jakus, 1995). Genetic abnormalities inactivating the *p16* gene might confer cell growth advantages contributing to the tumorigenic process.

*Received 8 July 1998*
*Revised 13 April 1999*
*Accepted 26 April 1999*

*Correspondence to:* MA Nagai

Genetic alterations involving the chromosomal region 9p21–22, such as translocations, insertions, heterozygous and homozygous deletions are frequently observed in human cancer. The *p16* gene is considered to be the deletion target in this region (Kamb et al, 1994*b*; Williamson et al, 1995). High frequencies of homozygous deletion and mutations of this gene have been detected in cell lines derived from different types of tumours (glioma, breast cancer, melanoma, lung, bladder, leukaemia) (Kamb et al, 1994*a*; Nobori et al, 1994), suggesting that *p16* may play an important role in the regulation of cellular growth in the majority of cells. However, in primary tumours, *p16* genetic alterations occur frequently in only a subset of tumour types (Koh et al, 1995; Pollock et al, 1996). The highest frequencies of *p16* inactivation by mutations and homozygous deletions are observed in carcinomas of the pancreas, oesophagus, renal cell, head and neck and in melanoma (Caldas et al, 1994; Mori et al, 1994; Cairns et al, 1995; Flores et al, 1996; Reed et al, 1996). Furthermore, germline p16 mutations predispose to familial melanoma (Hussussian et al, 1994; Kamb et al, 1994*b*).

Several studies have demonstrated high frequencies of loss of heterozygosity (LOH) on the short arm of chromosome 9 compared to the p16 mutations found in primary tumours. In addition, a complex pattern of LOH on 9p21–22 has been observed in different types of tumours, suggesting that this region may harbour other tumour suppressor genes associated with the tumorigenic process (Puig et al, 1995; Farrell et al, 1997; Kim et al, 1997). On the other hand, de novo methylation has been proposed to be an important alternative mechanism of *p16* gene inactivation. Merlo et al (1995), studying cell lines and primary solid tumours

(carcinomas of the lung, head and neck and gliomas), demonstrated that *p16* hypermethylation is a common event in those tumours. Subsequent studies have confirmed that *p16* is hypermethylated in carcinomas of the breast (31%), colon (40%), gliomas (44%), oesophageal adenocarcinomas (38%) and multiple myeloma (75%) (Herman et al, 1995; Fueyo et al, 1996; Lo et al, 1996; Ng et al, 1997; Wong et al, 1997).

Chromosome 9p deletions are considered to play a role in the early stages of the tumorigenic process of the head and neck (Califano et al, 1996). High frequencies of LOH of the 9p21–22 chromosomal region have been reported in squamous cell carcinomas of the head and neck, including dysplasia and carcinoma in situ (Nawroz et al, 1994; van der Reit et al, 1994). Analysis of *p16* mutations, hypermethylation and homozygous deletions showed that 7–79% of squamous cell carcinomas of the head and neck had at least one of those genetic events (Cairns et al, 1994; Zhang et al, 1994; Lydiatt et al, 1995; Reed et al, 1996; Jares et al, 1997); however, none of these studies have examined the biallelic inactivation of the *p16* and its relationship with the patients clinicopathological characteristics or survival.

In this report, to investigate the role of the *p16* genetic alterations in head and neck tumours, we performed a comprehensive analysis of the mechanisms involved in *p16* inactivation, such as mutations, hypermethylation, homozygous and heterozygous deletions. We further investigated whether there was a relationship between *p16* inactivation and clinicopathological characteristics and survival of the patients.

### MATERIALS AND METHODS

### Tissue samples

Paired tumour and normal tissue were obtained from 47 patients with primary head and neck squamous cell carcinoma, before any treatment, at the AC Camargo Hospital, São Paulo, Brazil. Tumours consisted of squamous cell carcinomas localized to the oral cavity ($n$ = 25), oropharynx ($n$ = 8), hypopharynx ($n$ = 7) and larynx ($n$ = 7). Tumour samples were dissected to remove residual normal tissue before freezing and storage in liquid nitrogen. To determine the amount of residual normal tissue, sections of tumour were stained with haematoxylin and eosin for histopathological examination. The amount of normal cell contamination in each tumour sample was estimated by the pathologist to not exceed 25%. The age of the patient at the time of operation ranged from 27 to 80 years (median 61). The study included a total of 40 males and seven females. Information on smoking history and alcohol intake was available from 36 and 31 patients respectively. Regular alcohol intake was declared by 83% of the smokers. The clinical stage of the patients was determined according to the UICC TNM staging system and histopathological grade based on the WHO classification.

### DNA extraction

Tissue was ground to a powder using a Frozen Tissue Pulverizer (Termovac), the powder was resuspended in 1 ml of lysis buffer (10 mM Tris–HCl, pH 7.6, 1 mM EDTA and 0.6% sodium dodecyl sulphate (SDS) and 100 μg $ml^{-1}$ proteinase K, and incubated at 37° overnight. High molecular weight DNA was extracted with phenol–chloroform and precipitated with ethanol.

### LOH analysis

LOH for the chromosomal region 9p21–22 was analysed using two polymerase chain reaction (PCR)-based polymorphic markers, as described previously (Kwiatkowsky and Diaz, 1993; Gyapay et al, 1994). Allelic losses were determined by densitometric scan (UltroScan XL; Pharmacia) as complete or partial if the intensity of one allele was reduced by at least 40% in tumour DNA as compared with normal DNA of the same patient. LOH was scored for informative (heterozygous) patients only.

### PCR – single-strand conformation polymorphism analysis

Two sets of oligonucleotide primers were used to amplify exons 1 and 2 of the *p16* gene, the primers used were the same as those described by Okamoto et al. (1994) and Sun et al (1995). PCR reactions were performed in 25-μl volumes using 50–100 ng of genomic DNA template, 1 μM of each primer, 1.5 mM magnesium chloride, 200 μM of each deoxynucleotide triphosphate, 0.1 μCi of [$\alpha^{32}$P-dCTP] (Amersham, specific activity, 3000 Ci $mmol^{-1}$), 50 mM potassium chloride, 10 mM Tris–HCl pH 8.0, and 0.5 unit of *Taq* DNA polymerase (Pharmacia, NJ, USA). Samples were overlaid with mineral oil and amplified for 35 cycles of denaturation, annealing and extension optimized for each primer set. The reactions were performed with an automated Thermal Cycler (Perkin-Elmer 580). Amplification products (1 μl) were diluted tenfold in a buffer containing 95% formamide, 20 mM EDTA, 0.05% bromophenol blue and 0.05% xylene cyanol, heated at 83°C for 5 min and applied (3 μl per lane) on two 6% polyaclamide non-denaturing gel, one containing 5% glycerol and the other 10% glycerol.

Electrophoresis was performed at 6 W for 14–16 h at room temperature with two cooling fans. Band shift mobility was detected by autoradiography of dried gels using Kodak X-Omat XAR film with an intensifying screen for 12–48 h at –70°C.

### Direct DNA sequencing

DNA samples with suspected *p16* mutations as judged by single-strand conformation polymorphism (SSCP) gels were amplified using the same primers. The PCR products obtained were purified using Wizard PCR Preps kit (Promega Corporation, Madison, WI, USA) according to the manufacturer's procedure. Three to 5 μl of ten out of the purified DNA was subjected to a dideoxy chain termination reaction using a double-stranded DNA Cycle Sequencing kit (Pharmacia) for both sense and antisense primers. Sequencing reaction products were denatured and resolved on 6% denaturing urea/polyacrylamide gels. Gels were fixed for 15 min in a 10% methanol/ 10% acetic acid solution, dried and exposed to X-ray film overnight.

### Homozygous deletion analysis

By Southern blot: high molecular weight DNAs (10 μg) were digested with *Eco*RI restriction endonuclease according to manufacturer's specification. Digested DNA samples were electrophoresed in 0.8% agarose gels with ethidium bromide and transferred to nylon membranes, which were hybridized with the PE1 probe described by Merlo et al (1995) labelled with [$^{32}$P]dCTP by random priming. Membrane hybridizations and

© 1999 Cancer Research Campaign

**Table 1** p16 genetic alterations observed in head and neck tumours

| Analyses | | Number of cases analysed | Alterations observed |
|---|---|---|---|
| PCR-SSCP | Exon 1 | 47 | 2/47 (6%) |
| | Exon 2 | 47 | 4/47 (8%) |
| Methylation assay | Exon 1 | 47 | 22/47 (47%) |
| LOH | IFNA | 47 | 11/28 (39%) |
| | D9S171 | 47 | 14/27 (52%) |
| Southern blot | | 47 | 1 case |

washing were performed as described previously (Nagai et al, 1993). Southern blots were stripped of probe by sodium hydroxide treatment and reprobed with a β-microglobulin probe to evaluate the amount of DNA loaded onto each lane. Scanning densitometry of the autoradiographies was carried out to quantify the signal intensity of the hybridized bands using an UltroScan XL (Pharmacia). By PCR, the same primers used for the SSCP analysis were used to investigate the occurrence of homozygous deletions. PCR reactions were performed using 100 ng of genomic DNA in the same conditions described for the SSCP analysis but with 24 cycles. Genomic DNA from the breast cancer cell line MCF-10F was used as positive control for homozygous deletion. PCR products were analysed on a 2% agarose gel.

### PCR-methylation assay

*p16* methylation status was examined using the combination of digestion of genomic DNA with methylation-sensitive restriction enzymes and PCR amplification. Genomic DNA (1 μg) was digested with 10 units of *Hpa*II, *Cfo*I or *Sma*I overnight according to manufacturer's instructions. In order to ensure complete digestion this step was repeated. Digested DNA samples were amplified by PCR using primers specific for exon 1 of the *p16* gene (Kamb et al, 1994*a*) and for a microsatellite marker (D9S145, 9q13–21.2), used as PCR control (Furlong et al, 1992). PCR was performed under the same conditions described for the SSCP analysis, without [$\alpha^{32}$P-dCTP], for 35 cycles of 94°C for 1 min, 55°C for 1 min, 72°C for 1 min and a final extension at 72°C for 5 min. The PCR products were analysed by electrophoresis on 2% agarose gels.

### Statistical methods

Analyses of statistical significance between the *p16* genetic alterations, and the clinicopathological characteristics of the patients were performed by the $\chi^2$ test and Fisher exact test for frequency data in contingency tables. Disease-free survival and overall survival probabilities were calculated based on the Kaplan–Meier product limit technique (Kaplan and Meier, 1958).

### RESULTS

Paired normal and tumour DNA from 47 patients with head and neck cancer were examined for the occurrence of *p16* genetic alterations (Table 1). Exons 1 and 2 of p16 were analysed for mutations by PCR-SSCP. Seven out of the 47 cases analysed showed evidence for *p16* mutations (exon 1, three cases; exon 2, four cases). DNA samples showing electrophoretic band shift mobility were re-amplified and the product purified and used directly for sequencing. Sequencing revealed the presence of six mutations and one intronic polymorphism. Figure 1 shows representative example of the sequencing analysis. Sequencing results are summarized in Table 2. The *p16* mutations observed included three mis-sense mutations (exon 1, codon 16, CTG→CCA, Leu→Pro; exon 2, codon 78, CTC→CAC, Leu→His; and exon 2, codon 114, CCC→CTC, Pro→Leu), one frameshift mutation (exon 2, codon 85, 1 bp insertion), one non-sense mutation (exon 2, codon 80, CGA→TGA, Arg→Stop) and one intronic mutation (intron 1, G→T; splicing alteration). All tumours with mutations were advanced stage tumours (two stage III and four stage IV), two in the oral cavity, two in the larynx and two in the hypopharynx.

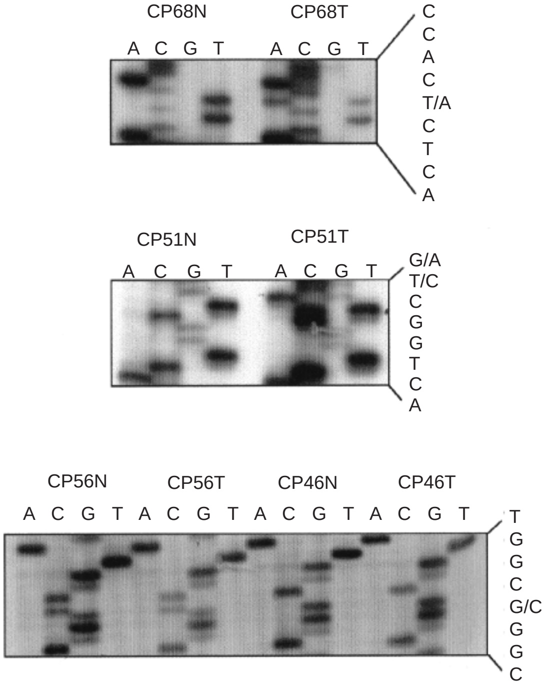

**Figure 1** Sequencing analysis of *p16* exons 1 and 2 mutations in head and neck tumours. Case CP68 and CP51 showed a mis-sense mutation in exon 2 (codon 78, CTC→CAC, Leu→His) and in exon 1 (codon 16, CTG→CCA, Leu→Pro) respectively. Case CP56 shows an intronic polymorphism

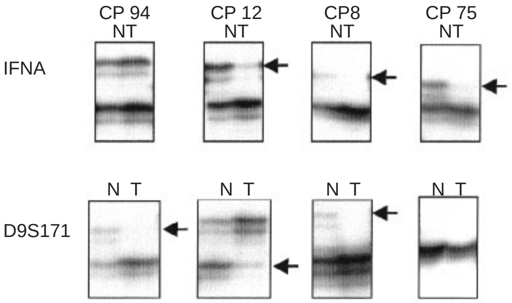

**Figure 2** Representative autoradiographs from loss of heterozygosity analysis of chromosome 9p in head and neck tumours. DNAs extracted from tumour (T) and corresponding normal (N) tissues were analysed using microsatellite markers IFNA and D9S171 as indicated on the left of the autoradiographs. Top, case numbers; arrow, allele showing reduction in intensity

© 1999 Cancer Research Campaign

**Table 2** Summary of the p16 mutations observed in head and neck tumours

| Case | Exon | Codon | Mutation | Effect |
|---|---|---|---|---|
| CP 1 | Intronl | | G→T | Splicing alteration |
| CP 16 | 2 | 80 | CGA→TGA | Arg→Stop |
| CP 28 | 2 | 85 | 1 bp insertion | Frameshift |
| CP 30 | 2 | 114 | CCC→CTC | Pro→Leu |
| CP 51 | 1 | 16 | CTG→CCA | Leu→Pro |
| CP 68 | 2 | 78 | CTC→CAC | Leu→His |

The occurrence of homozygous deletions was investigated by Southern blotting and PCR. Only one tumour DNA sample showed reduction (40%) in the intensity of the bands in the autoradiograms when compared with the corresponding normal DNA (data not shown), suggesting the occurrence of homozygous deletion.

LOH was analysed by PCR using two microsatellite markers, IFNA and D9S171, flanking the p16 locus (CDKN2). IFNA and D9S171 showed allelic loss in 11/28 (39%) and 14/27 (52%) informative cases respectively. Of the 32 informative tumours examined 17 (53%) showed LOH for at least one of the markers analysed. Representative results of the LOH analysis are shown in Figure 2.

Methylation status of the CpG sites in exon 1 of the *p16* gene was examined using methylation-sensitive enzymes (*Hpa*II, *Sma*I and *Cfo*I) and PCR amplification. Hypermethylation was detected in 22 of 47 cases analysed (47%). Tumours with different patterns of DNA methylation are shown in Figure 3. The absence of a 310 bp PCR product for exon 1 of the *p16* gene indicates that the *Hpa*II, *Sma*I and/or *Cfo*I restriction sites were unmethylated and had been cleaved (case CP44T). However, the presence of the 310 bp PCR product resistant to digestion with methylation-sensitive enzymes indicates the occurrence of de novo methylation (cases CP13T and CP88T).

In the present study, taking in account only the informative patients, *p16* biallelic inactivation was found in 59% (19/32) of the head and neck tumours analysed; 13 cases with hypermethylation and LOH; four cases with mutation and LOH; and two informative cases with retention of heterozygosity showing concomitant hypermethylation and mutation. In addition, homozygous deletion was observed in one case.

In the series of tumours examined no correlations were found between *p16* genetic alterations (mutation, hypermethylation and LOH together or alone) and age, tumour site, TNM staging,

**Table 3** Associations between p16 biallelic inactivation and the clinicopathological characteristics of 47 patients with head and neck tumours

| | | biallelic inactivation | | | |
|---|---|---|---|---|---|
| **Characteristics** | **Categories** | **N** | **No** | **Yes** | ***P*-value**[a] |
| Age | ≤ 50 years | 9 | 5 | 4 | |
| | > 50 years | 38 | 25 | 13 | 0.57 |
| Gender | Male | 40 | 25 | 15 | |
| | Female | 7 | 5 | 2 | 0.65 |
| Tumour site | Oral cavity | 24 | 16 | 8 | |
| | Oropharynx | 8 | 4 | 4 | |
| | Hypopharynx | 7 | 4 | 3 | |
| | Larynx | 7 | 5 | 2 | 0.75 |
| Lymph-node status | Negative | 22 | 13 | 9 | |
| | Positive | 25 | 17 | 8 | 0.53 |
| Histological grade[b] | I | 31 | 19 | 12 | |
| | II | 10 | 7 | 3 | |
| | III | 6 | 4 | 2 | 0.87 |
| Tumour stage[c] | I | 1 | – | 1 | |
| | II | 5 | 3 | 2 | |
| | III | 20 | 15 | 5 | |
| | IV | 21 | 12 | 9 | 0.31 |
| Tobacco consumption | Smoker | 36 | 23 | 13 | |
| | Non-smoker | 6 | 5 | 1 | 0.33 |
| Alcohol consumption | Drinker | 31 | 20 | 11 | |
| | Non-drinker | 10 | 8 | 2 | 0.35 |

[a]Chi-square test; [b]UICC TNM staging system; [c]WHO classification.

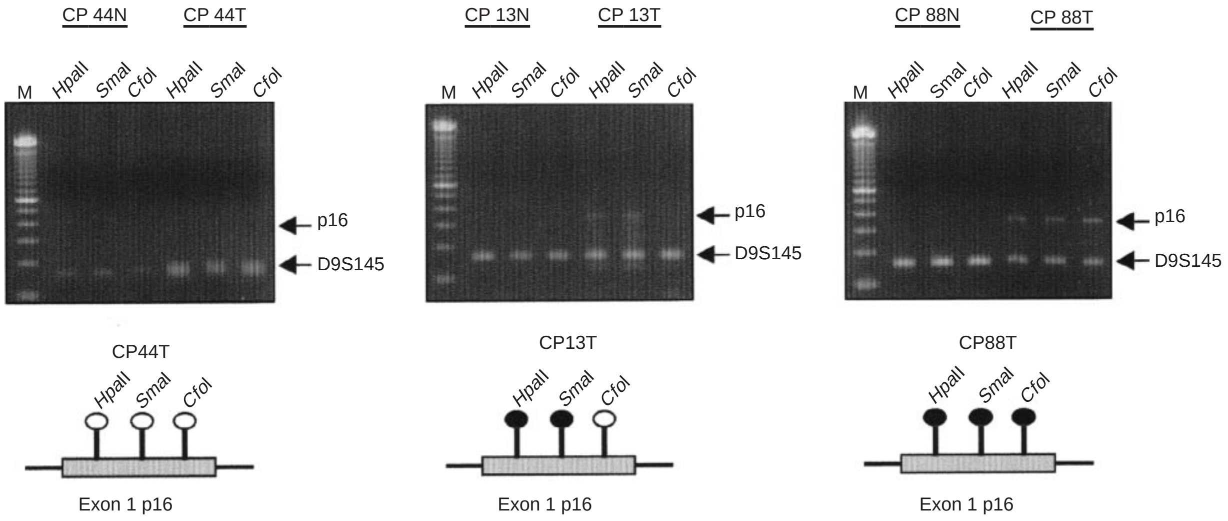

**Figure 3** Analysis of methylation status of the CpG island in exon 1 of the *p16* gene in head and neck tumours. The presence of a 310 bp PCR product after digestion with *Hpa*II, *Sma*I or *Cfo*I indicates de novo methylation. A representation of the methylation status of the restriction sites in each case is shown on the right. Case CP13T showed methylation at the *Hpa*II and *Sma*I sites; Case CP88T showed methylation at *Hpa*II, *Sma*I and *Cfo*I sites; and Case CP44T showed complete digestion at all restriction sites examined indicating absence of methylation

*© 1999 Cancer Research Campaign*

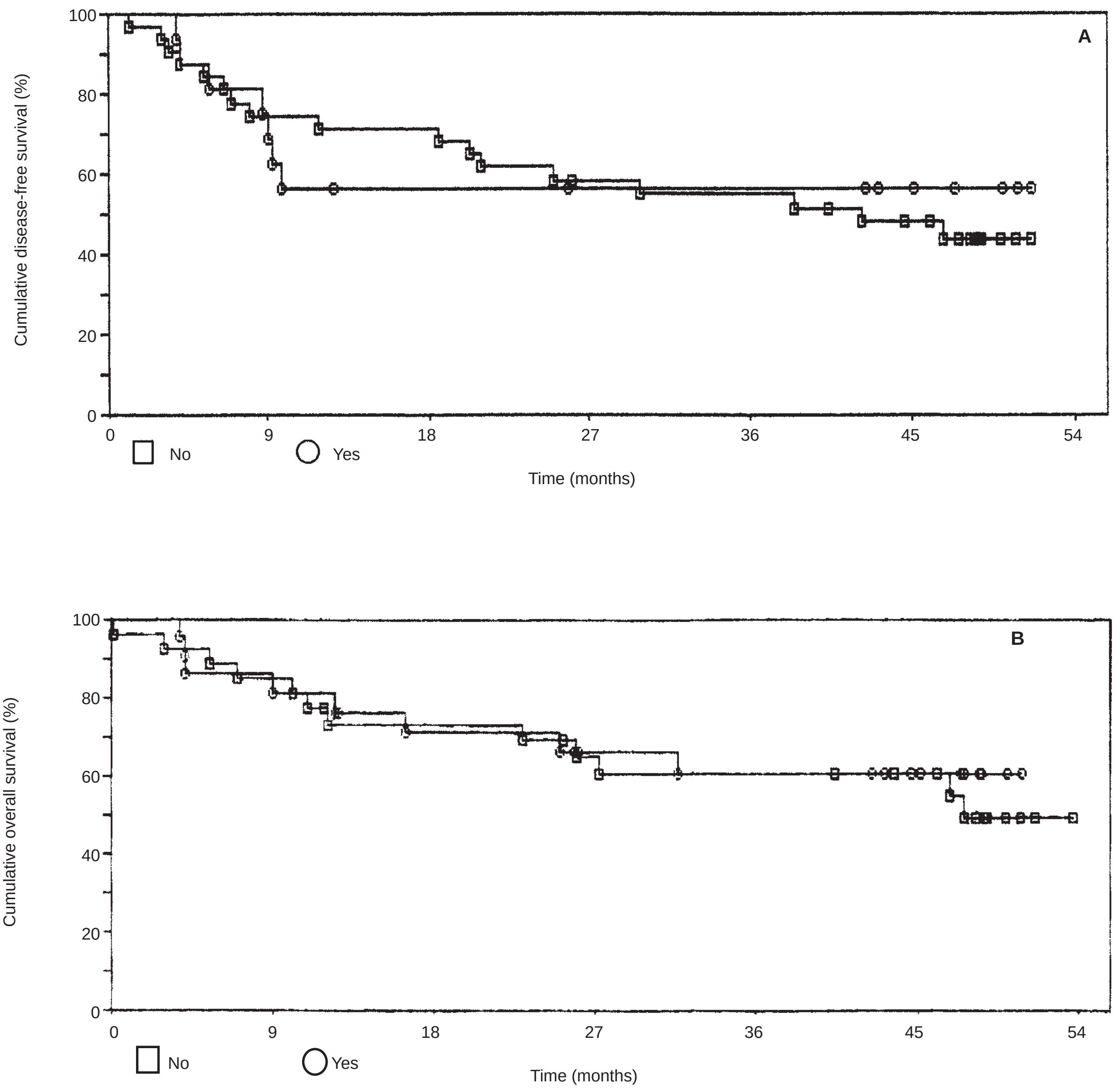

**Figure 4** Kaplan–Meier estimates of disease-free (**A**) and overall (**B**) survival in head and neck patients stratified according to the *p16* biallelic inactivation. □, patients with tumours showing p16 biallelic inactivation; ○, patients with tumours without *p16* biallelic inactivation (**A**, $P = 0.74$; **B**, $P = 0.73$)

histological differentiation, positive lymph nodes or tobacco and alcohol consumption of the patients (Table 3). In addition, no differences in survival were found between patients stratified for *p16* hypermethylation or biallelic inactivation (median survival 36.71 months) (Figure 4).

### DISCUSSION

Mutations, homozygous and heterozygous deletions and hypermethylation are the most common genetic events associated with the *p16* tumour suppressor gene inactivation. In the present study, we found evidence of *p16* inactivation in a high proportion (59%) of the head and neck tumours examined.

Thirteen per cent of the tumours analysed were found to have *p16* mutations. The base substitutions in exon 2 were at codons 78, 80 and 114 located within the ankyrin domains (Serrano et al, 1993). Mutations at codon 114 involved a highly conserved proline (P114) in the fourth ankyrin domain. Hence, the affected codons involve amino acids in domains which are likely to be essential for *p16* biological activity. Although, the exon 2 is shared by $p16^{ink4a}$ and $p19^{arf}$ (Serrano et al, 1996; Chin et al, 1998) experimental evidence indicates that mutations at exon 2 of the CDKN2A affects $p16^{ink4a}$ only (Arap et al, 1997). In addition, exon 1β of the CDKN2A appears to be critical for $p19^{arf}$ function, both binding of $p19^{arf}$ to p53 and cell cycle arrest requires exon 1β but not exon 2 (Quelle et al, 1997; Kamijo et al, 1998). Furthermore, in the present study we have observed a point mutation in the acceptor site of intron 1 and a missense mutation in exon 1α (codon 16) implicating $p16^{ink4a}$ as the major target of inactivation in the head and neck tumours analysed.

*© 1999 Cancer Research Campaign*

Although homozygous deletions has been reported to be an important mechanism of *p16* inactivation in several human cancers, including head and neck tumours (Reed et al, 1996), in our series of tumours only one tumour showed evidence for homozygous deletion. Despite the fact that Southern blot and PCR analyses were used to examine the occurrence of homozygous deletions, we cannot rule out the possibility that normal cell contamination could account for the exquisitely low frequency of homozygous deletion found in the present study.

LOH and DNA hypermethylation were observed in 53% and 47% of the cases analysed respectively, representing the major mechanisms which may lead to *p16* inactivation. In total 59% (19/32) of the informative patients with head and neck tumours examined showed evidence of *p16* biallelic inactivation. Overall, 20% (4/20) of the cases with biallelic inactivation showed mutation and hypermethylation, 10% (2/20) showed mutations and LOH, 65% (13/20) showed hypermethylation and LOH and 5% (1/20) showed homozygous deletion. These observations indicate that LOH and hypermethylation leading to p16 inactivation is common in head and neck tumours. These results are similar to those of Wong et al (1997) in oesophageal adenocarcinomas and corroborate with previous studies that have demonstrated high incidence of p16 hypermethylation in head and neck tumours (El-Naggar et al, 1997; Gonzáles et al, 1997).

To assess the prognostic potential of p16 inactivation in the development of head and neck tumours the genetic alterations observed (alone and in combination) were correlated with the clinicopathological characteristics (such as age, tumour size, lymph node status, clinical stage, histological grade) and patient outcome. Our study failed to demonstrate any correlation between *p16* inactivation and these clinicopathological characteristics or survival of the patients. LOH of chromosomal region 9p21 has been postulated to be an early event in head and neck cancer (Califano et al, 1996) and *p16* inactivation has been detected in preneoplastic lesions of the larynx and oral cavity (Gallo et al, 1997; Papadimitrakopolou et al, 1997). Our failure to find prognostic significance for the *p16* genetic alterations might suggest that *p16* inactivation is an early event in carcinogenesis in a subgroup of head and neck tumours but with little or no influence on further tumour progression. The high frequence of tumours with *p16* biallelic inactivation observed here provides further support to previous report (Reed et al, 1996) that *p16* tumour suppressor gene does play an important role in the tumorigenic process of the head and neck. This hypothesis is also supported by observations that *p16* expression inhibits growth in cell lines derived from squamous cell carcinomas of the head and neck (Liggett et al, 1996). However, whether *p16* inactivation is an important predictor for prognosis and disease outcome needs to be clarified by further molecular epidemiological studies.

### ACKNOWLEDGEMENTS

We are grateful to Dr Lois M Mulligan for critical review of this manuscript. This work was supported by a grant from CNPq/PADCT 62.0097/94.9.

### REFERENCES

Arap W, Knudsen E, Sewell DA, Sidransky D, Wang JY, Huang HJ and Cavenee WK (1997) Functional analysis of wild type and malignant glioma derived CDKN2Abeta alleles: evidence for an Rb-independent growth supressive pathway. *Oncogene* **15**: 2013–2020

Cairns P, Mao L, Merlo A, Lee DJ, Schwab D, Eby Y, Tokino K, van der Riet P, Blaugrund JE and Sidransky D (1994) Rates of *p16* (*MTS1*) mutations in primary tumors with 9p loss. *Science* **265**: 415–416

Cairns P, Polascik TJ, Eby Y, Tokino K, Califano J, Merlo A, Mao L, Herath J, Jenkins R, Westra W, Rutter JL, Buckler A, Gabrielson E, Tockman M, Cho KR, Hedrick L, Bova GS, Isaacs W, Koch W, Schwab D and Sidransky D (1995) Frequency of homozygous deletion at p16/CDKN2 in primary human tumours. *Nat Genet* **11**: 210–212

Caldas C, Hahn SA, Costa LT, Redston MS, Schutte M, Seymour AB, Weinstein CL, Hruban RH, Yeo CJ and Kern SE (1994) Frequent somatic mutations and homozygous deletions of the *p16* (*MTS1*) gene in pancreatic adenocarcinoma. *Nat Genet* **8**: 27–32

Califano J, van der Riet P, Westra W, Nawroz H, Clayman G, Piantadosi S, Corio R, Lee D, Greenberg B, Koch W and Sidransky D (1996) Genetic progression model for head and neck cancer: implications for field cancerization. *Cancer Res* **56**: 2488–2492

Chin L, Pomerantz J and DePinho RA (1998) The INK4a/ARF tumor supressor: one gene, two products, two pathways. *Trends Biochem. Sci* **23**: 291–296

El-Naggar AK, Lai S, Clayman G, Lee JK, Luna MA, Goepfert H and Batsakis JG (1997) Methylation, a major mechanism of p16/cdkn2 gene inactivation in head and neck squamous carcinoma. *Am J Pathol* **151**: 1767–1774

Farrell WE, Simpson DJ, Bicknell JE, Talbot AJ, Bates AS and Clayton RN (1997) Chromosome 9p deletions in invasive and noninvasive non-functional pituitary adenomas: the deleted region involves markers outside of the MTS1 and MTS2 genes. *Cancer Res* **57**: 2703–2709

Flores JF, Walker GJ, Glendening JM, Haluska FG, Castresana JS, Rubio MP, Pastorfide GC, Boyer LA, Kao WH, Bulyk ML, Barnhill RL, Hayward NK, Housman DE and Fountain JW (1996) Loss of $p16^{INK4a}$ and $p15^{INK4b}$ genes, as well as neighboring 9p21 markers, in sporadic melanoma. *Cancer Res* **56**: 5023–5032

Fueyo J, Gomez-Manzano C, Bruner JM, Saito Y, Zhang B, Zhang W, Levin VA, Yung WA and Kyritsis AP (1996) Hypermethylation of the CpG island of *p16/cdkn2* correlates with gene inactivation in gliomas. *Oncogene* **13**: 1615–1619

Furlong RA, Lyall JE, Lush MJ, Affara NA and Ferguson-Smith MA (1992) Four dinucleotide repeat polymorphisms on chromosome 9 (D9S143-D9S146). *Hum Mol Genet* **1**: 447

Gallo O, Santucci M and Franchi A (1997) Cumulative prognostic value of *p16/cdkn2* and *p53* oncoprotein expression in premalignant laryngeal lesions. *J Natl Cancer Inst* **89**: 1161–1163

Gonzáles MV, Pello MF, López-Larrea C, Suárez C, Menéndez MJ and Coto E (1997) Deletion and methylation of the tumour supressor gene *p16/cdkn2* in primary head and neck squamous cell carcinoma. *J Clin Pathol* **50**: 509–512

Gyapay G, Morissete J, Vignal A, Dib C, Fizames C, Millasseau P, Marc S, Bernardi G, Lanttrop M and Weissenbach J (1994) The 1993–1994 généthon human genetic linkage map. *Nat Genet* **7**: 246–339

Herman JG, Merlo A, Mao L, Lapidus RG, Issa JPJ, Davidson NE, Sidransky D and Baylin SB (1995) Inactivation of the *cdkn2/p16/MTS1* gene is frequently associated with aberrant DNA methylation in all common human cancers. *Cancer Res* **55**: 4525–4530

Hunter T and Pines J (1994) Cyclins and cancer II: cyclin D and CDK inhibitors come of age. *Cell* **79**: 573–582

Hussussian CJ, Struewing JP, Goldstein AM, Higgins PAT, Ally DS, Sheahan MD, Clark WH, Tucker MA and Dracopoli NC (1994) Germline p16 mutations in familial melanoma. *Nat Genet* **8**: 15–21

Jares P, Fernández PL, Nadal A, Cazorla M, Hernández L, Pinyol M, Hernández S, Traserra J, Cardesa A and Campo E (1997) $p16^{MTS1/CDK41}$ mutations and concomitant loss of heterozygosity at 9p21–23 are frequent events in squamous cell carcinoma of the larynx. *Oncogene* **15**: 1445–1453

Jarrard DF, Bova GS, Ewing CM, Pin SS, Nguyen SH, Baylin SB, Cairns P, Sidransky D, Herman JG and Isaacs WB (1997) Deletional, mutational and methylation analyses of CDKN2 (p16/MST1) in primary and metastatic prostate cancer. *Genes Chromosomes Cancer* **19**: 90–96

Kamb A, Gruis NA, Weaver-Feldhaus J, Liu Q, Harshman K, Tavtigian SV, Stockert E, Day III RS, Johnson BE and Skolnick MH (1994*a*). A cell cycle regulator potentially involved in genesis of many tumor types. *Science* **264**: 436–440

Kamb A, Shattuck-Eidens D, Eeles R, Liu Q, Gruis NA, Ding W, Hussey C, Tran T, Miki Y, Weaver-Feldhaus J, McClure M, Aitken JF, Anderson DE, Bergman W, Frants R, Goldgar DE, Green A, MacLennan R, Martin NG, Meyer LJ, Youl P, Zone JJ, Skolnick MH and Cannon-Albright LA (1994*b*). Analysis of the p16 gene (CDKN2) as a candidate for the chromosome 9p melanoma susceptibility locus. *Nat Genet* **8**: 22–26

© 1999 Cancer Research Campaign

Kamijo T, Weber JD, Zambetti G, Zindy F, Roussel MF, Sherr CJ (1998) Functional and physical interactions of the ARF tumor supressor with p53 and Mdm2. *Proc Natl Acad Sci USA* **95**: 8292–8297

Kaplan EL and Meier P (1958) Non-parametric estimation from incomplete observations. *J Am Stat Assoc* **53**: 457–481

Kim SK, Ro JY, Kemp BL, Lee JS, Kwon TJ, Fong KM, Sekido Y, Minna JD, Hong WK and Mao L (1997) Identification of three distinct tumor suppressor loci on the short arm of chromosome 9 in small cell lung cancer. *Cancer Res* **57**: 400–403

Koh J, Enders GH, Dynlacht BD and Harlow E (1995) Tumor-derived p16 alleles encoding proteins defective in cell-cycle inhibition. *Nature* **375**: 506–510

Kwiatkowski DJ and Diaz MO (1993) Dinucleotide repeat polymorphism at IFNA locus (9p22). *Hum Mol Genet* **1**: 658

Liggett WH Jr, Sewell DA, Rocco J, Ahrendt SA, Koch W and Sidransky D (1996) p16 and p16β are potent growth suppressors of head and neck squamous carcinoma cells in vitro. *Cancer Res* **56**: 4119–4123

Lo KW, Cheung ST, Leung SF, van Hasselt A, Tsang YS, Mak KF, Shung YF, Woo JKS, Lee JCK and Huang DP (1996) Hypermethylation of p16 gene in nasopharyngeal carcinoma. *Cancer Res* **56**: 2721–2725

Lydiatt WM, Murty VVVS, Davidson BJ, Xu L, Dyomina K, Sacks PG, Schantz SP and Chaganti RSK (1995) Homozygous deletions and loss of expression of the CDKN2 gene occur frequently in head and neck squamous cell carcinoma cell lines but infrequently in primary tumors. *Genes Chrom & Cancer* **13**: 94–98

Maesawa C, Tamura G, Nishizuka S, Ogasawara S, Ishida K, Terashima M, Sakata K, Sato N, Saito K and Satodate R (1996) Inactivation of the CDKN2 gene by homozygous deletion and de Novo methylation is associated with advanced stage esophageal squamous cell carcinoma. *Cancer Res* **56**: 3875–3878

Merlo A, Herman JG, Mao L, Lee DJ, Gabrielson E, Burger PC, Baylin SB and Sidransky D (1995) 5′CpG island methylation is associated with transcriptional silencing of the tumor suppressor p16/CDKN2/MTS1 in human cancers. *Nat Med* **1**: 686–692

Mori T, Miura K, Aoki T, Nishihira T, Mori S and Nakamura Y (1994) Frequent somatic mutation of MTS1/CDKN4I (multiple tumor suppressor/cyclin dependent kinase 4 inhibitor) gene in esophageal squamous cell carcinoma. *Cancer Res* **54**: 3396–3397

Nagai MA, Marques LA, Torloni H and Brentani MM (1993) Genetic alterations in c-erbB-2 protooncogene as prognostic markers in human primary breast tumors. *Oncology* **50**: 412–417

Nawroz H, van der Riet P, Hruban RH, Koch W, Ruppert JM and Sidransky D (1994) Allelotype of head and neck squamous cell carcinoma. *Cancer Res* **54**: 1152–1155

Ng MHL, Chung YF, Lo KW, Wickham NWR, Lee JCK and Huang DP (1997) Frequent hypermethylation of p16 and p15 genes in multiple myeloma. *Blood* **89**: 2500–2506

Nobori T, Miura K, Wu DJ, Lois A, Takabayashi K and Carson DA (1994) Deletions of the cyclin-dependent kinase-4 inhibitor gene in multiple human cancers. *Nature* **368**: 753–756

Okamoto A, Demetrick DJ, Spillare EA, Hagiwara K, Hussain SP, Bennett WP, Forrester K, Gerwin B, Serrano M, Beach DH and Harris CC (1994) Mutations and altered expression of p16 in human cancer. *Proc Natl Acad Sci USA* **91**: 11045–11049

Papadimitrakopoulou V, Izzo J, Lippman SM, Lee JS, Fan YH, Clayman G, Ro JY, Hittelman WN, Lotan R, Hong WK and Mao L (1997) Frequent inctivation $p16^{INK4a}$ in oral premalignant lesions. *Oncogene* **14**: 1799–1803

Pollock PM, Pearson JV and Hayward NK (1996) Compilation of somatic mutations of the CDKN2 gene in human cancers: non-random distribution of base substitutions. *Genes Chromosomes Cancer* **15**: 77–88

Puig S, Ruiz A, Lázaro C, Castel T, Lynch M, Palou J, Vilalta A, Weissenbach J, Mascaro JM and Estivill X (1995) Chromosome 9p deletions in cutaneous malignant melanoma tumors: the minimal deleted region involves markers outside de p16 (CDKN2) gene. *Am J Hum Genet* **57**: 395–402

Quelle DE, Cheng M, Ashmun RA and Sherr CJ (1997) Cancer-associated mutations at the INK4a locus cancel cell cycle arrest by $p16^{INK4a}$ but not by the alternative reading frame protein $p19^{ARF}$. *Proc Natl Acad Sci USA* **94**: 669–673

Quelle DE, Zindy F, Ashmun RA and Sherr CJ (1995) Alternative reading frames of the INK4a tumor supressor gene encode two unrelated proteins capable of inducing cell cycle arrest. *Cell* **83**: 993–1000

Ranade K, Hussussian CJ, Sikorski RS, Varmus HE, Goldstein AM, Tucker MA, Serrano M, Hannon GJ, Beach D and Dracopoli NC (1995) Mutations associated with familial melanoma impair p16 (INK4) function. *Nat Genet* **10**: 114–116

Reed AL, Califano J, Cairns P, Westra WH, Jones RM, Koch W, Ahrendt S, Eby Y, Sewell D, Nawroz H, Bartek J and Sidransky D (1996) High frequency of p16 (CDKN2/MTS-1/INK4A) inactivation in head and neck squamous cell carcinoma. *Cancer Res* **56**: 3630–3633

Serrano M, Hannon GJ and Beach D (1993) A new regulatory motif in cell cycle control causing specific inhibition of cyclin D/CDK4. *Nature* **366**: 704–707

Serrano M, Lee H, Chin L, Cordon-Cardo C, Beach D, DePinho RA (1996) Role of INK4a locus in tumor supression and cell mortality. *Cell* **85**: 27–37

Stone S, Jiang P, Dayananth P, Tavtigian SW, Katcher H, Parry D, Peters G and Kamb A (1995) Complex structure and regulation of the p16 (MTS1) locus. *Cancer Res* **55**: 2988–2994

Sun Y, Hildesheim A, Lanier AE, Cao Y, Yao KT, Raab-Traub N and Yang CS (1995) No point mutation but decreased expression of p16/MTS1 tumor suppressor gene in nasopharyngeal carcinomas. *Oncogene* **10**: 785–788

Tam SW, Shay JW and Pagano M (1994) Differential expression and cell cycle regulation of the cyclin-dependent kinase 4 inhibitor $p16^{INK4}$. *Cancer Res* **54**: 5816–5820

van der Reit P, Nawroz H, Hruban RH, Corio R, Tokino K, Koch W and Sidransky D (1994) Frequent loss of chromosome 9p21–22 early in head and neck progression. *Cancer Res* **54**: 1156–1158

Williamson MP, Elder PA, Shaw ME, Devlin J and Knowles MA (1995) p16 (CDKN2) is a major deletion target at 9p21 in bladder cancer. *Hum Mol Genet* **4**: 1569–1577

Wong DJ, Barret MT, Stöger R, Emond MJ and Reid BJ (1997) p16INK4a promoter is hypermethylated at high frequency in esophageal adenocarcinomas. *Cancer Res* **57**: 2619–2622

Yeundall WA and Jakus J (1995). Cyclin kinase inhibitors add a new dimension to cell cycle control. *Eur J Cancer B Oral Oncol* **31B**: 291–298

Zhang SY, Klein-Szanto AJP, Sauter ER, Shafarenko M, Mitsunaga S, Nobori T, Carson DA, Ridge JA and Goodrow TL (1994) Higher frequency of alterations in the p16/CDKN2 gene in squamous cell carcinoma cell lines than in primary tumours of the head and neck. *Cancer Res* **54**: 5050–5053

*© 1999 Cancer Research Campaign*

## References

[PDF_00597] Arap W., Knudsen E., Sewell D. A., Sidransky D., Wang J. Y., Huang H. J., Cavenee W. K. (1997). Functional analysis of wild-type and malignant glioma derived CDKN2Abeta alleles: evidence for an RB-independent growth suppressive pathway.. Oncogene.

[PDF_00601] Cairns P., Mao L., Merlo A., Lee D. J., Schwab D., Eby Y., Tokino K., van der Riet P., Blaugrund J. E., Sidransky D. (1994). Rates of p16 (MTS1) mutations in primary tumors with 9p loss.. Science.

[PDF_00604] Cairns P., Polascik T. J., Eby Y., Tokino K., Califano J., Merlo A., Mao L., Herath J., Jenkins R., Westra W. (1995). Frequency of homozygous deletion at p16/CDKN2 in primary human tumours.. Nat Genet.

[PDF_00609] Caldas C., Hahn S. A., da Costa L. T., Redston M. S., Schutte M., Seymour A. B., Weinstein C. L., Hruban R. H., Yeo C. J., Kern S. E. (1994). Frequent somatic mutations and homozygous deletions of the p16 (MTS1) gene in pancreatic adenocarcinoma.. Nat Genet.

[PDF_00613] Califano J., van der Riet P., Westra W., Nawroz H., Clayman G., Piantadosi S., Corio R., Lee D., Greenberg B., Koch W. (1996). Genetic progression model for head and neck cancer: implications for field cancerization.. Cancer Res.

[PDF_00617] Chin L., Pomerantz J., DePinho R. A. (1998). The INK4a/ARF tumor suppressor: one gene--two products--two pathways.. Trends Biochem Sci.

[PDF_00619] El-Naggar A. K., Lai S., Clayman G., Lee J. K., Luna M. A., Goepfert H., Batsakis J. G. (1997). Methylation, a major mechanism of p16/CDKN2 gene inactivation in head and neck squamous carcinoma.. Am J Pathol.

[PDF_00622] Farrell W. E., Simpson D. J., Bicknell J. E., Talbot A. J., Bates A. S., Clayton R. N. (1997). Chromosome 9p deletions in invasive and noninvasive nonfunctional pituitary adenomas: the deleted region involves markers outside of the MTS1 and MTS2 genes.. Cancer Res.

[PDF_00626] Flores J. F., Walker G. J., Glendening J. M., Haluska F. G., Castresana J. S., Rubio M. P., Pastorfide G. C., Boyer L. A., Kao W. H., Bulyk M. L. (1996). Loss of the p16INK4a and p15INK4b genes, as well as neighboring 9p21 markers, in sporadic melanoma.. Cancer Res.

[PDF_00633] Fueyo J., Gomez-Manzano C., Bruner J. M., Saito Y., Zhang B., Zhang W., Levin V. A., Yung W. K., Kyritsis A. P. (1996). Hypermethylation of the CpG island of p16/CDKN2 correlates with gene inactivation in gliomas.. Oncogene.

[PDF_00637] Furlong R. A., Lyall J. E., Lush M. J., Affara N. A., Ferguson-Smith M. A. (1992). Four dinucleotide repeat polymorphisms on chromosome 9 (D9S143-146).. Hum Mol Genet.

[PDF_00640] Gallo O., Santucci M., Franchi A. (1997). Cumulative prognostic value of p16/CDKN2 and p53 oncoprotein expression in premalignant laryngeal lesions.. J Natl Cancer Inst.

[PDF_00643] González M. V., Pello M. F., López-Larrea C., Suárez C., Menéndez M. J., Coto E. (1997). Deletion and methylation of the tumour suppressor gene p16/CDKN2 in primary head and neck squamous cell carcinoma.. J Clin Pathol.

[PDF_00647] Gyapay G., Morissette J., Vignal A., Dib C., Fizames C., Millasseau P., Marc S., Bernardi G., Lathrop M., Weissenbach J. (1994). The 1993-94 Généthon human genetic linkage map.. Nat Genet.

[PDF_00650] Herman J. G., Merlo A., Mao L., Lapidus R. G., Issa J. P., Davidson N. E., Sidransky D., Baylin S. B. (1995). Inactivation of the CDKN2/p16/MTS1 gene is frequently associated with aberrant DNA methylation in all common human cancers.. Cancer Res.

[PDF_00654] Hunter T., Pines J. (1994). Cyclins and cancer. II: Cyclin D and CDK inhibitors come of age.. Cell.

[PDF_00656] Hussussian C. J., Struewing J. P., Goldstein A. M., Higgins P. A., Ally D. S., Sheahan M. D., Clark W. H., Tucker M. A., Dracopoli N. C. (1994). Germline p16 mutations in familial melanoma.. Nat Genet.

[PDF_00659] Jares P., Fernández P. L., Nadal A., Cazorla M., Hernández L., Pinyol M., Hernández S., Traserra J., Cardesa A., Campo E. (1997). p16MTS1/CDK4I mutations and concomitant loss of heterozygosity at 9p21-23 are frequent events in squamous cell carcinoma of the larynx.. Oncogene.

[PDF_00664] Jarrard D. F., Bova G. S., Ewing C. M., Pin S. S., Nguyen S. H., Baylin S. B., Cairns P., Sidransky D., Herman J. G., Isaacs W. B. (1997). Deletional, mutational, and methylation analyses of CDKN2 (p16/MTS1) in primary and metastatic prostate cancer.. Genes Chromosomes Cancer.

[PDF_00668] Kamb A., Gruis N. A., Weaver-Feldhaus J., Liu Q., Harshman K., Tavtigian S. V., Stockert E., Day R. S., Johnson B. E., Skolnick M. H. (1994). A cell cycle regulator potentially involved in genesis of many tumor types.. Science.

[PDF_00680] Kamijo T., Weber J. D., Zambetti G., Zindy F., Roussel M. F., Sherr C. J. (1998). Functional and physical interactions of the ARF tumor suppressor with p53 and Mdm2.. Proc Natl Acad Sci U S A.

[PDF_00685] Kim S. K., Ro J. Y., Kemp B. L., Lee J. S., Kwon T. J., Fong K. M., Sekido Y., Minna J. D., Hong W. K., Mao L. (1997). Identification of three distinct tumor suppressor loci on the short arm of chromosome 9 in small cell lung cancer.. Cancer Res.

[PDF_00689] Koh J., Enders G. H., Dynlacht B. D., Harlow E. (1995). Tumour-derived p16 alleles encoding proteins defective in cell-cycle inhibition.. Nature.

[PDF_00691] Kwiatkowski D. J., Diaz M. O. (1992). Dinucleotide repeat polymorphism at the IFNA locus (9p22).. Hum Mol Genet.

[PDF_00693] Liggett W. H., Sewell D. A., Rocco J., Ahrendt S. A., Koch W., Sidransky D. (1996). p16 and p16 beta are potent growth suppressors of head and neck squamous carcinoma cells in vitro.. Cancer Res.

[PDF_00696] Lo K. W., Cheung S. T., Leung S. F., van Hasselt A., Tsang Y. S., Mak K. F., Chung Y. F., Woo J. K., Lee J. C., Huang D. P. (1996). Hypermethylation of the p16 gene in nasopharyngeal carcinoma.. Cancer Res.

[PDF_00699] Lydiatt W. M., Murty V. V., Davidson B. J., Xu L., Dyomina K., Sacks P. G., Schantz S. P., Chaganti R. S. (1995). Homozygous deletions and loss of expression of the CDKN2 gene occur frequently in head and neck squamous cell carcinoma cell lines but infrequently in primary tumors.. Genes Chromosomes Cancer.

[PDF_00704] Maesawa C., Tamura G., Nishizuka S., Ogasawara S., Ishida K., Terashima M., Sakata K., Sato N., Saito K., Satodate R. (1996). Inactivation of the CDKN2 gene by homozygous deletion and de novo methylation is associated with advanced stage esophageal squamous cell carcinoma.. Cancer Res.

[PDF_00708] Merlo A., Herman J. G., Mao L., Lee D. J., Gabrielson E., Burger P. C., Baylin S. B., Sidransky D. (1995). 5' CpG island methylation is associated with transcriptional silencing of the tumour suppressor p16/CDKN2/MTS1 in human cancers.. Nat Med.

[PDF_00712] Mori T., Miura K., Aoki T., Nishihira T., Mori S., Nakamura Y. (1994). Frequent somatic mutation of the MTS1/CDK4I (multiple tumor suppressor/cyclin-dependent kinase 4 inhibitor) gene in esophageal squamous cell carcinoma.. Cancer Res.

[PDF_00716] Nagai M. A., Marques L. A., Torloni H., Brentani M. M. (1993). Genetic alterations in c-erbB-2 protooncogene as prognostic markers in human primary breast tumors.. Oncology.

[PDF_00719] Nawroz H., van der Riet P., Hruban R. H., Koch W., Ruppert J. M., Sidransky D. (1994). Allelotype of head and neck squamous cell carcinoma.. Cancer Res.

[PDF_00722] Ng M. H., Chung Y. F., Lo K. W., Wickham N. W., Lee J. C., Huang D. P. (1997). Frequent hypermethylation of p16 and p15 genes in multiple myeloma.. Blood.

[PDF_00725] Nobori T., Miura K., Wu D. J., Lois A., Takabayashi K., Carson D. A. (1994). Deletions of the cyclin-dependent kinase-4 inhibitor gene in multiple human cancers.. Nature.

[PDF_00728] Okamoto A., Demetrick D. J., Spillare E. A., Hagiwara K., Hussain S. P., Bennett W. P., Forrester K., Gerwin B., Serrano M., Beach D. H. (1994). Mutations and altered expression of p16INK4 in human cancer.. Proc Natl Acad Sci U S A.

[PDF_00732] Papadimitrakopoulou V., Izzo J., Lippman S. M., Lee J. S., Fan Y. H., Clayman G., Ro J. Y., Hittelman W. N., Lotan R., Hong W. K. (1997). Frequent inactivation of p16INK4a in oral premalignant lesions.. Oncogene.

[PDF_00736] Pollock P. M., Pearson J. V., Hayward N. K. (1996). Compilation of somatic mutations of the CDKN2 gene in human cancers: non-random distribution of base substitutions.. Genes Chromosomes Cancer.

[PDF_00739] Puig S., Ruiz A., Lázaro C., Castel T., Lynch M., Palou J., Vilalta A., Weissenbach J., Mascaro J. M., Estivill X. (1995). Chromosome 9p deletions in cutaneous malignant melanoma tumors: the minimal deleted region involves markers outside the p16 (CDKN2) gene.. Am J Hum Genet.

[PDF_00743] Quelle D. E., Cheng M., Ashmun R. A., Sherr C. J. (1997). Cancer-associated mutations at the INK4a locus cancel cell cycle arrest by p16INK4a but not by the alternative reading frame protein p19ARF.. Proc Natl Acad Sci U S A.

[PDF_00748] Quelle D. E., Zindy F., Ashmun R. A., Sherr C. J. (1995). Alternative reading frames of the INK4a tumor suppressor gene encode two unrelated proteins capable of inducing cell cycle arrest.. Cell.

[PDF_00751] Ranade K., Hussussian C. J., Sikorski R. S., Varmus H. E., Goldstein A. M., Tucker M. A., Serrano M., Hannon G. J., Beach D., Dracopoli N. C. (1995). Mutations associated with familial melanoma impair p16INK4 function.. Nat Genet.

[PDF_00755] Reed A. L., Califano J., Cairns P., Westra W. H., Jones R. M., Koch W., Ahrendt S., Eby Y., Sewell D., Nawroz H. (1996). High frequency of p16 (CDKN2/MTS-1/INK4A) inactivation in head and neck squamous cell carcinoma.. Cancer Res.

[PDF_00759] Serrano M., Hannon G. J., Beach D. (1993). A new regulatory motif in cell-cycle control causing specific inhibition of cyclin D/CDK4.. Nature.

[PDF_00761] Serrano M., Lee H., Chin L., Cordon-Cardo C., Beach D., DePinho R. A. (1996). Role of the INK4a locus in tumor suppression and cell mortality.. Cell.

[PDF_00763] Stone S., Jiang P., Dayananth P., Tavtigian S. V., Katcher H., Parry D., Peters G., Kamb A. (1995). Complex structure and regulation of the P16 (MTS1) locus.. Cancer Res.

[PDF_00766] Sun Y., Hildesheim A., Lanier A. E., Cao Y., Yao K. T., Raab-Traub N., Yang C. S. (1995). No point mutation but decreased expression of the p16/MTS1 tumor suppressor gene in nasopharyngeal carcinomas.. Oncogene.

[PDF_00769] Tam S. W., Shay J. W., Pagano M. (1994). Differential expression and cell cycle regulation of the cyclin-dependent kinase 4 inhibitor p16Ink4.. Cancer Res.

[PDF_00776] Williamson M. P., Elder P. A., Shaw M. E., Devlin J., Knowles M. A. (1995). p16 (CDKN2) is a major deletion target at 9p21 in bladder cancer.. Hum Mol Genet.

[PDF_00779] Wong D. J., Barrett M. T., Stöger R., Emond M. J., Reid B. J. (1997). p16INK4a promoter is hypermethylated at a high frequency in esophageal adenocarcinomas.. Cancer Res.

[PDF_00782] Yeudall W. A., Jakus J. (1995). Cyclin kinase inhibitors add a new dimension to cell cycle control.. Eur J Cancer B Oral Oncol.

[PDF_00784] Zhang S. Y., Klein-Szanto A. J., Sauter E. R., Shafarenko M., Mitsunaga S., Nobori T., Carson D. A., Ridge J. A., Goodrow T. L. (1994). Higher frequency of alterations in the p16/CDKN2 gene in squamous cell carcinoma cell lines than in primary tumors of the head and neck.. Cancer Res.

[PDF_00773] van der Riet P., Nawroz H., Hruban R. H., Corio R., Tokino K., Koch W., Sidransky D. (1994). Frequent loss of chromosome 9p21-22 early in head and neck cancer progression.. Cancer Res.

